# An Image-Based High-Content Screening Assay for Compounds Targeting Intracellular *Leishmania donovani* Amastigotes in Human Macrophages

**DOI:** 10.1371/journal.pntd.0001671

**Published:** 2012-06-12

**Authors:** Jair L. Siqueira-Neto, Seunghyun Moon, Jiyeon Jang, Gyongseon Yang, Changbok Lee, Hong Kee Moon, Eric Chatelain, Auguste Genovesio, Jonathan Cechetto, Lucio H. Freitas-Junior

**Affiliations:** 1 Center for Neglected Diseases Drug Discovery (CND3), Institut Pasteur Korea, Seongnam-si, Gyeonggi-do, South Korea; 2 Image Mining Group, Institut Pasteur Korea, Seongnam-si, Gyeonggi-do, South Korea; 3 Screening Technology & Pharmacology Group, Institut Pasteur Korea, Seongnam-si, Gyeonggi-do, South Korea; 4 Drugs for Neglected Diseases initiative (DNDi), Geneva, Switzerland; René Rachou Research Center, Brazil

## Abstract

Leishmaniasis is a tropical disease threatening 350 million people from endemic regions. The available drugs for treatment are inadequate, with limitations such as serious side effects, parasite resistance or high cost. Driven by this need for new drugs, we developed a high-content, high-throughput image-based screening assay targeting the intracellular amastigote stage of different species of *Leishmania* in infected human macrophages. The *in vitro* infection protocol was adapted to a 384-well-plate format, enabling acquisition of a large amount of readouts by automated confocal microscopy. The reading method was based on DNA staining and required the development of a customized algorithm to analyze the images, which enabled the use of non-modified parasites. The automated analysis generated parameters used to quantify compound activity, including infection ratio as well as the number of intracellular amastigote parasites and yielded cytotoxicity information based on the number of host cells. Comparison of this assay with one that used the promastigote form to screen 26,500 compounds showed that 50% of the hits selected against the intracellular amastigote were not selected in the promastigote screening. These data corroborate the idea that the intracellular amastigote form of the parasite is the most appropriate to be used in primary screening assay for *Leishmania*.

## Author Summary

Leishmaniasis, one of the most neglected tropical diseases, affects over 2 million people each year. Visceral leishmaniasis (VL), also known as Kala-azar, is caused by the protozoan parasites *Leishmania donovani* and *Leishmania infantum* and is fatal if left untreated. Because existing treatments are often ineffective due to parasite resistance and/or toxicity new drugs are urgently needed. Leishmaniasis is transmitted to humans by the bite of an infected sandfly. In the insect vector, parasites exist as flagellated forms—promastigotes, which infect macrophage cells of the human host, where they differentiate to round forms known as amastigotes. Amastigotes and promastigotes are substantially different from a molecular perspective. Drug discovery for leishmaniasis has traditionally been complicated by the unavailability of validated drug targets and of relevant drug assays. Whole cell-based assays have been widely used, as they bypass the need for a validated target. However, they use the insect form of the parasite; indeed, the human form, the intracellular amastigote, is difficult to obtain in the laboratory in quantities compatible with drug screening. We describe here the technical advances that made it possible to adapt the intracellular amastigote form of *L. donovani* to a drug assay compatible with high-throughput screening.

## Introduction

Leishmaniasis is a tropical disease caused by parasites of the genus *Leishmania*, with clinical manifestations ranging from localized cutaneous ulcers to systemic visceral organ damage. The visceral form of the disease is the most severe and is lethal if not treated. The organs targeted by the parasites are determined mainly by the infecting parasite species and the patient's immune system. In the human host, the parasite is able to infect different cell types, with macrophages as the final host in which the parasites differentiate from promastigotes into amastigotes and multiply [1]. Leishmaniasis is endemic to 88 countries in tropical and sub-tropical areas and threatens 350 million people [2].

There is no available vaccine [3], and the drugs used for treatment have major drawbacks, including parasite resistance and high toxicity, with strong side effects for the patient [4], [5]. New drugs or formulations are therefore urgently needed [6], [7]. High-throughput screening (HTS) is an efficient way of identifying active compounds among large numbers of small molecules, thereby feeding drug discovery pipelines with new candidates and optimizing both research costs and time [8]. High-content screening (HCS) combines the efficiency of HTS with information-rich assays to provide several measures of a compound's effect in the assay system. The requirements for HCS assays include quantifiable and reproducible measurements of compound activity compared to standard reference drugs in concentrations achievable in serum/tissues, handling of ultra-small (nanogram) amounts of compound, and adaptation of the assay to standard microplate formats and laboratory automation platforms.

Here we report the development and validation of a protocol for *in vitro* drug screening and automated image mining for leishmaniasis using the intracellular amastigote form of *Leishmania* to infect human macrophages. We applied an image-based approach and developed computer-assisted algorithms to interpret the infection and quantify the activities of the anti-parasitic compounds. Other assays in medium- to high-throughput format have already been developed for anti-leishmanial drug screening using promastigotes (insect forms) or axenic amastigotes [9], [10], [11], [12]. While these forms of the parasite are easier to adapt to an HTS assay format, promastigotes are not representative of the human disease, and the use of axenic amastigotes as a model of intracellular amastigotes is controversial because amastigotes are exclusively intracellular *in vivo* [13]. To confirm the importance of the parasite stage used in an HTS assay with a large number of compounds, we compared screening data for a subset of 26,500 compounds obtained using either this newly developed assay or a previously developed HTS assay that used the promastigote form of the parasite.

## Materials and Methods

### Parasites and cells

*Leishmania donovani* MHOM/ET/67/HU3, *Leishmania amazonensis* MHOM/BR/73/M2269, *Leishmania braziliensis* MHOM/BR/2903 and *Leishmania major* MHOM/IL/81/FRIEDLIN promastigotes were axenically cultivated at 28°C in 199 Culture Medium (Sigma M5017) with 40 mM Hepes (Gibco 15630), 0.1 mM adenine (Sigma A5251), 0.0001% biotin (Sigma B329) and 4.62 mM NaHCO_3_ (Sigma S5761), supplemented with 10% (or 20% for *L. braziliensis*) heat-inactivated fetal bovine serum (FBS, Gibco 16000) and 1% streptomycin/penicillin (Gibco 15140). The cultures were diluted every 3 or 4 days to maintain the parasite density between 10^6^ parasites/ml and 4×10^7^ parasites/ml. To avoid generation of genetic variability, we kept the parasite for a maximum of 20 sub-cultured dilution cycles, thawing new vials from the same frozen stock. THP-1, the human acute leukemia monocyte cell line (ATCC TIB-202), was cultivated in RPMI medium (Gibco 61870-036) supplemented with 10% heat-inactivated FBS (Gibco 16000) and 1% streptomycin/penicillin (Gibco 15140) at 37°C and 5% CO_2_. The cultures were diluted every 3 or 4 days to maintain the cell density between 10^5^ cells/ml and 8×10^5^ cells/ml. Cells were kept for a maximum of 20 sub-cultured dilution cycles, thawing new vials from the same frozen stock.

### Infection

The *Leishmania* culture at a density of 10^6^ parasites/ml was incubated for 6 days before infection to enrich the proportion of metacyclic promastigotes. THP-1 cells at 5×10^5^ cells/ml were differentiated with 50 ng/ml of phorbol 12-myristate 13-acetate (PMA, Sigma P1585) for 48 hours at 37°C, 5% CO_2_. Differentiated THP-1 cells are adherent and were seeded at a confluence of 1.8×10^5^ cells/cm^2^. Trypsinized THP-1 cells were mixed with the 6-day-old *Leishmania* promastigotes at a final density of 4×10^5^ THP-1/ml and 2×10^7^ parasites/ml in RPMI medium supplemented with 10% FBS. This homogeneous mixture of differentiated THP-1 cells and parasites was seeded in 384-well plates at 50 µl/well using a WellMate (Thermo Scientific) liquid handler and incubated for 5 days at 37°C, 5% CO_2_.

### Intramacrophagic *Leishmania* amastigote replication assay

To visualize amastigote replication, differentiated THP-1 cells were seeded on coverslip slides in 24-well plates (8×10^4^ cells/well) and infected with 4×10^6^ parasites in a final volume of 200 µl RPMI supplemented with 10% FBS. The cells were washed 3 times with PBS 12 hours after parasite addition. To detect DNA replication, 5-bromo-2-deoxyuridine (BrdU) was used. A mixture of 1 mM BrdU and 1 mM deoxycytidine (dC) was added to the infected THP-1 culture and incubated for 12 hours. The cultures were then washed with PBS, fixed with cold 100% methanol for 5 minutes and air dried for 5 minutes at room temperature. Next, 1.5 M HCl prepared in PBS was added to the samples and incubated for 15 minutes, followed by PBS washing and permeabilization with 0.1% Triton X-100 in PBS for 10 minutes. After another PBS wash, the cells were incubated for 1 hour at 4°C with mouse anti-BrdU conjugated monoclonal antibody (Invitrogen, 1∶400 dilution) in PBS containing 4% BSA, washed 3 times with PBS and further incubated for 1 hour at 4°C with Alexa Fluor® 488 goat anti-mouse IgG (Invitrogen, 1∶400 dilution), 2 µM DAPI (Sigma D9564) and 2.5 µM Syto60 (Invitrogen MOP-S-11342) in PBS containing 4% BSA. The samples were washed 3 times with PBS and mounted over slides with Vecta-shield (Vector H-1000). Staining was visualized with a Leica confocal TCS SP2 fluorescence microscope, taking pictures in different focal planes (z-stack). Imaris (Bitplane) software was used to develop the 3D model after acquisition of a series of images in different focal planes.

### Reference compounds, nuclei staining and fluorescence microscopy

The anti-leishmanial reference drugs used were amphotericin B (Sigma A9528), miltefosine (Merck 475841), paromomycin sulfate (Sigma P9297) and sodium stibogluconate (Sigma S5319). The reference drugs and tested compounds were added 24 hours after infection and incubated at 37°C and 5% CO_2_ for 4 days. The cells and parasites were then fixed with 2% paraformaldehyde and stained with 5 µM Draq5 (Biostatus DR50200) in PBS. Fluorescent images (4 images per well) were acquired from each assay well using an Opera confocal microscope (Perkin Elmer) with a 635 nm laser at 20× lens magnification.

### Data analysis (software development)

The acquired images were analyzed with an Image Mining (IM) platform developed in-house. The IM software directly accessed the databases of image acquisition platforms and created a flow of images, which were sequentially analyzed by dedicated algorithms developed as plugins of the software. The results of all analyses were stored in a centralized database.

**Figure 1:**
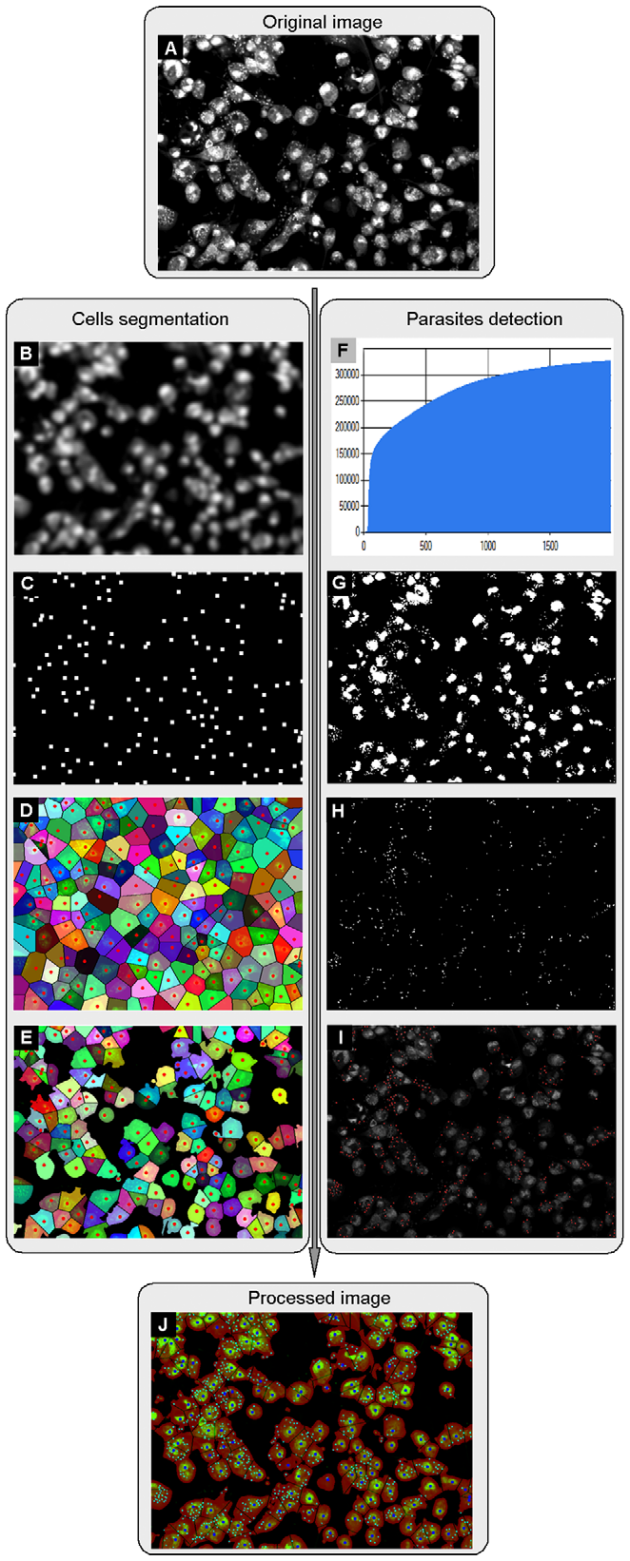
Parameters analyzed for automated image analysis and infection level measurement. **A**) **Input image:** Raw image input acquired with Opera confocal microscope. **B–E**) **Cell segmentation based on nuclei detection.** **B**) Image denoising by a Gaussian kernel of radius 5. **C**) Local maxima point detection from B to define nuclei positions. **D**) Voronoi diagram computation based on the nuclei positions to delineate the inner boundaries of the attached cells. **E**) Threshold cut-off of pixels below a selected intensity level to make the foreground mask. This image is an example of final cell segmentation. **F–I**) **Parasite detection.** **F**) Calculation of the upper 50% cumulative intensity level of the raw image (A). **G**) Threshold cut-off of pixels below the intensity level of (F). **H**) Objects smaller than 4 or larger than 15 pixels are removed to classify parasites. **I**) Parasite positions are defined. **J**) **Result image:** the merged images of cell segmentation (E) and parasite detection (I).

Using a simple DNA staining technique to permit the use of wild-type parasites without any reporter gene was interesting because its quantification relied solely on the accuracy of the image analysis, and it permitted various parasite species to be used without the need for genetic manipulation. The DNA staining proved to be a simple and stable cell marker, and the image analysis software plugin that was developed took into account both the accuracy and speed constraints of HCS. Several methods were tested, and some steps were necessary to detect cells and parasites to obtain a robust separation between the positive and negative controls. Fig. 1 presents a flow chart of those steps.

The quantification of the infection ratio consisted of cell segmentation based on nuclei and parasite detection components. The two components were processed independently from the same raw input image (Fig. 1A) and merged to identify individual cells and assign detected parasites to each cell object (Fig. 1J).

The cell segmentation was as follows (Fig. 1B–E): the raw input image was smoothed with a Gaussian kernel [14] of standard deviation 5, which roughly corresponds to the radius of the nucleus (Fig. 1B). Then, the local extreme detection method [15] was applied to the smoothed image to extract local maxima points, which indicated the number and positions of nuclei (Fig. 1C). Subsequently, the inner boundaries of the individual cells were identified by computing the Voronoi diagram [16] on the foreground area using the previously obtained set of local maxima as seed points (Fig. 1D). Finally, a foreground mask (Fig. 1E) was applied to the Voronoi diagram image to remove background area. For the parasite detection (Fig. 1F–I), the algorithm calculated the higher 50% cumulative intensity level of the raw input image (Fig. 1F), used a threshold cut-off in the image based on the intensity level to remove background area and the cytoplasm of cells to isolate nuclei and parasites (Fig. 1G) and, finally, used the connected component labeling method [17] to filter out nuclei objects and other artifacts by detecting as parasites the connected component objects that were simultaneously larger than 4 pixels and smaller than 15 pixels (Fig. 1I). The final result from a processed image is shown (Fig. 1J), with cell detection and segmentation merged with parasite detection.

**Figure 2:**
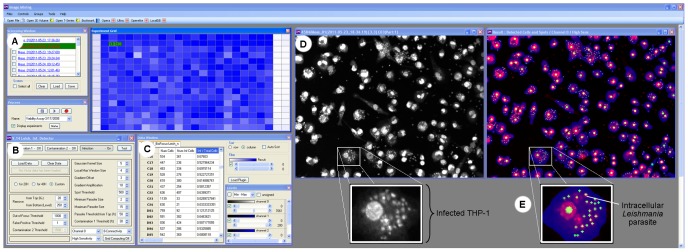
Software interface. **A**) List of plates in the left and graphical representation of the infection ratio of a 384-well plate by gradient color intensity: white is 0% infection, and blue is 100% infection, with the colors in between indicating an intermediate infection ratio. The first two columns carry controls of amphotericin B EC_100_-treated wells and non-treated wells (1% DMSO), respectively. The last two columns (23 and 24) are non-infected wells and contain non-infected THP-1 cells. **B**) The window control to set up the software parameters for optimal tuning. **C**) Table of parameters generated by the software analysis, including, from left to right, “total number of cells,” “number of infected cells” and “infection ratio.” **D**) A raw image at left and colors highlighting element detection after software analysis: blue (THP-1 cells), red (nuclei of THP-1 detected cells) and green (parasites). **E**) Close-up of a selected infected THP-1 from (D), highlighting the intracellular *Leishmania* parasite.

The IM software interface is shown in Fig. 2. A color-based graphical representation of the 384-well plate enabled a quick visual analysis of the results. Fig. 2A illustrates one DMSO control plate, highlighting the infection ratio as the scaling factor for the blue color. Algorithm parameters may be modified to optimize the analysis of the images as shown in the window illustrated in Fig. 2B. The software provides 7 output features that can be used for statistical analysis (Fig. 2C). These features include the following: 1) number of cells; 2) number of infected cells (cells containing at least one parasite); 3) infection ratio (the number of infected cells divided by the number of cells); 4) total number of parasites per cell; 5) standard deviation of the number of parasites per cell; 6) total area of the cells (number of pixels occupied by cells), and 7) average area per cell (average number of pixels per cell). Fig. 2D–E shows images from the wells before (left) and after (right) IM analysis, highlighting the identification of THP-1 cells and *Leishmania* parasites.

### Data normalization and assay quality control

The calculated activity was normalized to percentage infection (Inf) based on the amphotericin B EC_100_ (effective concentration showing 100% activity, meaning minimum measured infection ratio) and 1% DMSO (0% activity, meaning maximum measured infection ratio) controls according to the formula: % infection = (measured IR−μ_AmpB EC100_)/(μ_1%DMSO_−μ_AmpB EC100_)×100, in which μ_1%DMSO_ is the average infection ratio of the 1% DMSO controls and μ_AmpB EC100_ is the average infection ratio of the amphotericin B EC_100_ control.

The quality of the assay data was primarily assessed with the Z' factor [18]. In addition to the Z' factor, a number of other parameters were taken into consideration to assess the robustness of the developed assay. These include the following: i) pharmacological validation as assessed by dose response curves for the reference drug amphotericin B. In the current assay, the EC_50_ for amphotericin B was determined to be approximately 0.3 µM, in agreement with previously published values [19]; ii) evaluation of any type of plate pattern such as edge effects, particularly important for assays with multi-day incubations, or other patterns introduced, for example, by liquid handling devices; iii) quantitative assessment of variability in the infection ratio, cell and parasite number on different days and with different batches of cells and parasites.

## Results

### Biological model development

**Figure 3:**
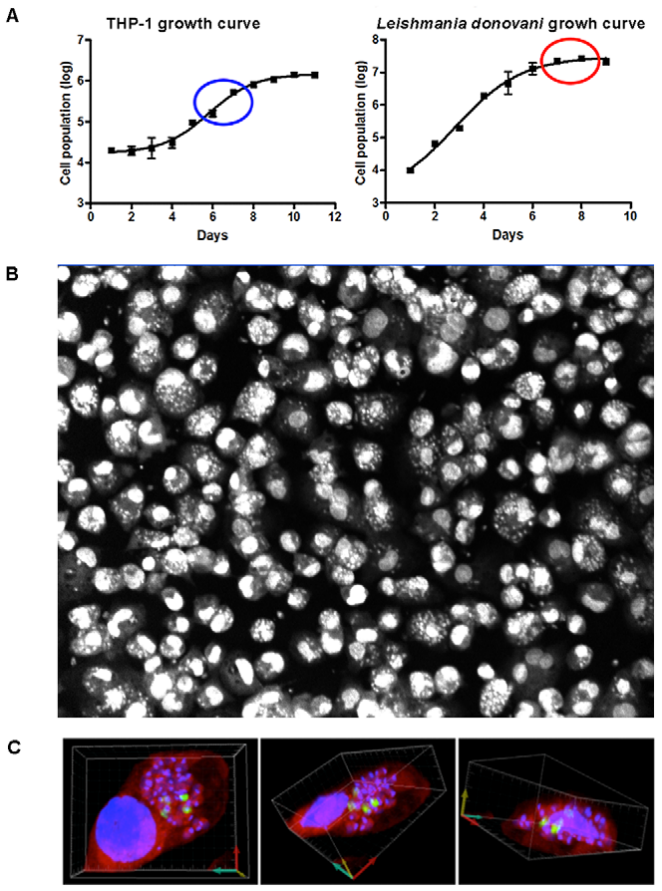
THP-1 infection with *L. donovani*. **A**) Growth curves of THP-1 and *L. donovani*, with the optimal development points for infection highlighted by blue and red circles, respectively. **B**) Image acquired with an Opera confocal microscope showing THP-1 infected with *L. donovani* after Draq5 (DNA) staining. **C**) 3-D reconstitution of multiple series confocal pictures illustrating from two different perspectives THP-1 macrophages stained with Syto-60 (red) and infected by *L. donovani* parasites. Dapi was used to stain the DNA (blue) of both the host cells and the parasites. BrdU incorporation detected by immunofluorescence (green) indicates the replication of intracellular amastigote parasites.

The screening assay was based on the use of late-stage promastigote *Leishmania* cultures to infect differentiated THP-1 macrophages and the quantification of the infection ratio 4 days after compound addition. THP-1 human macrophages have been used as a *Leishmania* host model for *in vitro* infection for over two decades [20], [21], [22] and have been proposed to be suitable for drug screening against the intracellular form of the parasite [12]. We investigated different time points in the growth curve of the THP-1 cells and *Leishmania* parasites to determine the optimal development stage for each cell line, in order to optimize the infection ratio. Fig. 3A illustrates the growth curves and highlights the optimal culture durations for the host cells and parasites prior to infection. The use of 5- or 6-day-old late-log-phase promastigote cultures resulted in higher infection ratios because of the enrichment of metacyclic promastigotes in the culture media, as previously reported [23]. A high infection ratio obtained with this protocol is illustrated in Fig. 3B, with an average of 88.7% (±4.7%).

Reference drugs and assay compounds were added to the plate 24 hours after infection. The protocol was intended to select compounds active against intracellular amastigotes. To demonstrate that within this time interval the late stage promastigotes had been phagocytized by macrophages and had differentiated into amastigotes within the macrophages, we visualized the intracellular replication of the parasite using a BrdU incorporation assay. The presence of incorporated BrdU, as detected by immunofluorescence, demonstrated that 24 hours after infection, the intracellular parasites were indeed differentiated into amastigotes and were replicating inside the host cell. This result validated the protocol of adding compounds 24 hours after infection to target intracellular amastigotes. The same replication assay was also performed 4 days after infection. Pictures were taken in different focal planes, and a 3D model was built, as shown in Movie S1. Fig. 3C shows snapshots of the 3D model. Parasites labeled in green indicate replicating parasites.

### Assay optimization and HTS adaptation

When high throughput is considered for biological experiments, the assay must be adapted to best fit the requirements of the large scale. However, to compare results from different laboratories, we developed a *Leishmania* infection standard protocol adapted to an HCS/HTS method, with the potential for implementation on a smaller laboratory scale without the need for automation. To define this standard protocol, several conditions were tested to find the highest and most reproducible infection ratio, using *L. donovani* parasites to define and validate the assay. The parasite containers were one of the tested variables, comparing a T175 flask with a 1.0 L Erlenmeyer bottle under agitation, being the latter the best option (Figure S1). We also tested the effect of PMA concentration and incubation time on THP-1 cell differentiation, as illustrated in Figure S2, showing that 50 ng/ml yielded best results. The method for releasing differentiated THP-1 cells was also important for the preservation and integrity of the cells, and different approaches were tested, being trypsinization the chosen method (Figure S3).

To evaluate the pharmacological relevance of the assay, we tested four different anti-leishmanial reference drugs in our system: amphotericin B, miltefosine, paromomycin and sodium stibogluconate. Dose-response curves (DRC) were determined in three independent experiments, with four replicates used to define the EC_50_s. The results for each reference drug are given in Table 1. The EC_50_s of all of the reference drugs were comparable to the reported values [19], [24], thus validating this new assay system for compound screening. Miltefosine is a known anti-cancer drug [25], and, at the EC_100_ dose, cytotoxicity was observed for the THP-1 host cell, an acute monocytic leukemia cell line. Sodium stibogluconate did not eliminate intracellular parasites in the experiment; this compound is known to be poorly active *in vitro*, and its slow effect was incompatible with the exposure time window for our experiment [23], [26]. Paromomycin was not efficient either in eliminating intracellular *L. donovani* from the THP-1 host; its activity was inconsistent, and it was inactive in some experiments, as previously reported [19]. This variability is not acceptable for HTS purposes. We chose amphotericin B as a reference drug in the screening assay because it had an EC_50_ in the target range for new active compounds (nanomolar), was stable, was not cytotoxic up to 20 µM and showed reproducible results. In addition, amphotericin B is a first-line drug used for the treatment of visceral leishmaniasis in many endemic countries [4].

**Table 1:** Anti-*L. donovani* reference drug activity.

	EC_50_ (s.d.)
Amphotericin B	0.3 µM (±0.2)
Miltefosine	3.1 µM (±2.3)
Paromomycin	>20 µM
Sodium stibogluconate	>100 µg/ml

### Assay automation and HTS validation

Because the assay was conducted with live parasites and host cells over multiple days, a fully automated (*i.e.*, unattended) protocol was not feasible. However, several time-consuming and repetitive steps in the assay were automated, enabling a potential throughput of ∼20,000 wells per screening day. An infection batch of host cells and parasites was added to the plates with a bulk dispenser (Wellmate, Thermo Scientific). The plates were then loaded onto the automated platform for compound addition, incubation and reading (all automated steps).

The first step in automation is the scale-up of the assay. In this case, cells and reagents were prepared at the same scale as the one to be used during an HTS campaign and compared to the results for a small scale preparation (Figure S4). Each of the liquid handling devices to be used in the automation process was individually validated for accuracy and reproducibility. The assay validation consisted of the simulation of 3 independent screening days using only control plates. Each run was composed of 20 plates, 10 DRC plates containing amphotericin B as the reference drug and 10 DMSO plates as the negative control. The validation was performed on a cell::explorer™ automated platform with an Opera confocal microscope (Perkin Elmer) for imaging.

**Figure 4:**
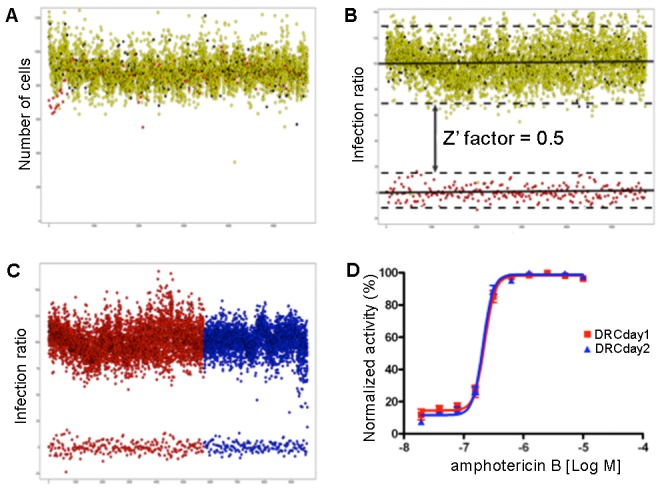
High-throughput screening validation. **A**) Plot of the number of cells (Y-axis) in the controls: yellow (1% DMSO - compound position in the plate), black (1% DMSO – control position in the plate) and red (amphotericin B EC_100_). **B**) Plot of the infection ratio (Y-axis) using the same color code as in A). The Z' factor of 0.5 demonstrates the statistical confidence of the assay. **C**) Plot of the infection ratios (Y-axis) obtained from different validation days (red representing day 1 and blue representing day 2) demonstrating low day-to-day variation in the infection ratio and a clear window between non-treated controls (1% DMSO in the upper portion of the plot) and amphotericin B EC_100_-treated controls (lower portion of the plot). **D**) DRCs of amphotericin B from two independent validation days (red and blue curves representing days 1 and 2, respectively), demonstrating the consistency in the anti-leishmanial activity of the reference drug.

The validation run using the semi-automated screening method and the results indicated that the assay was robust, both within a screening day and across screening days. Within a screening day, the number of THP-1 cells in both the DMSO controls and the amphotericin B (EC_100_) wells was similar, indicating that the cell number was consistent between the control and DMSO wells, within a plate and across multiple plates (Fig. 4A). Based on the infection ratio, there was a clear window between the DMSO controls and amphotericin B (Fig. 4B). This window was consistent throughout the validation day and across multiple validation days (Fig. 4C). The assay also showed a consistent EC_50_ in the range of 0.3 µM for the reference compound, amphotericin B, between screening days (Fig. 4D). The Z' factor within a validation day and across multiple days was 0.5, indicating that the assay was high quality. A flowchart of the entire screening methodology is illustrated in Figure S5.

**Figure 5:**
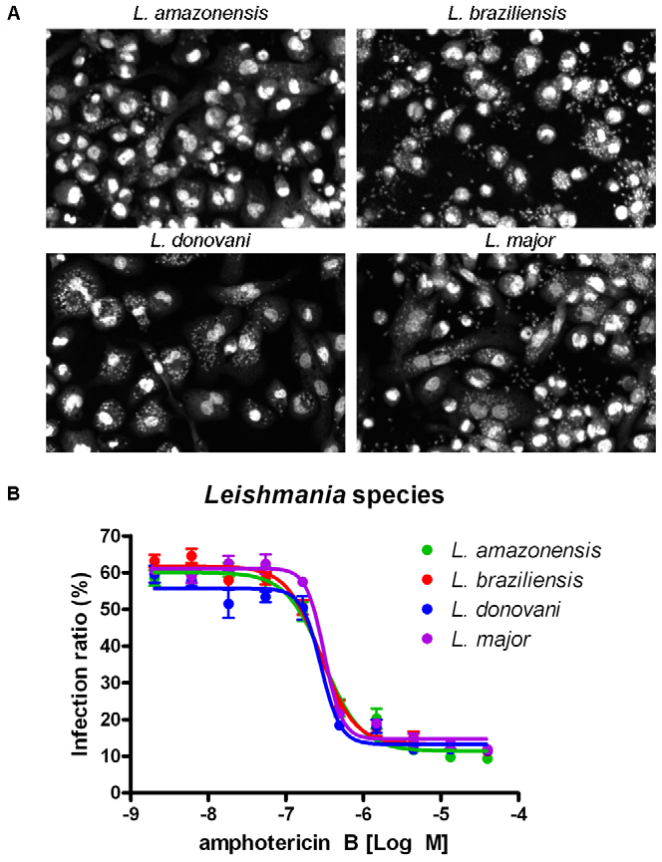
*In vitro* infection assay optimization for four *Leishmania* species. **A**) Images illustrating the infection of THP1 human macrophages by *Leishmania* species that causes diseases with different clinical manifestations: *L. amazonensis* (diffuse cutaneous), *L. braziliensis* (mucocutaneous), *L. donovani* (visceral) and *L. major* (cutaneous). **B**) Dose response curves for the reference drug amphotericin B against all four species.

To determine the specificity of the compounds, we used the same assay principle with species of *Leishmania* other than *L. donovani*, which cause different clinical manifestations. We used *L. major*, *L. amazonensis* and *L. braziliensis* as representatives of cutaneous and mucocutaneous leishmaniasis. Images illustrating the infection of THP-1 cells with these 3 species in addition to *L. donovani* are depicted in Fig. 5A. The DRCs for amphotericin B demonstrated that all of the species had similar *in vitro* sensitivities, with the following EC_50_s: 0.30 µM for *L. amazonensis*, 0.28 µM for *L. braziliensis*, 0.28 µM for *L. donovani* and 0.31 µM for *L. major* (Fig. 5B).

We previously published an HTS screen with *Leishmania* promastigote forms [12], and here we evaluated the correlation between the results from that promastigote-based screen and the amastigote-based screen (this work). We used a library of 26,500 compounds and evaluated the outputs from each approach. The screen using *L. major* promastigotes with compounds at 10 µM generated 124 hits after a 70% activity cut-off (3 standard deviations from the negative control average) and the exclusion of compounds that interfered with the growth of non-differentiated THP-1 (potentially toxic), with only 5 compounds (4%) showing activity against intracellular *L. major* amastigotes at concentrations up to 20 µM [12]. An independent screen was performed using the same compound library (26,500) at 20 µM against *L. donovani* using the new infection assay described in this study. The number of hits based on a 55% activity cut-off (3 standard deviations from the negative control average), no cytotoxicity against differentiated THP-1 (based on cell counting) and an EC_50_<20 µM was 123 compounds (Figure S6), coincidently almost the same number of hits selected in the promastigote screening. From the 123 hit compounds, 62 showed activity against promastigotes. We also confirmed these data, in which only 51% of the hits obtained from the intracellular amastigotes (24 compounds out of 47 hits) were also active against the promastigote form, by screening a focused library of 4,000 kinase and phosphatase inhibitors using *L. donovani* (data not shown). The results from the 26,500 compounds are illustrated in Figure S6 and provide evidence that performing screens with intracellular amastigote forms would increase the probability of finding compounds active against the human form of the parasite (amastigote) and would be the most appropriate way to find compounds that exclusively target the intracellular form that infects the host cell.

## Discussion

HTS technology has been primarily associated with target-based assays. This approach requires substantial efforts prior to screening to identify and validate a target involved in the disease process. Once a target is identified and validated, compounds can be screened against it to identify inhibitors in either biochemical (e.g., purified enzyme) assays or cell-based (e.g., receptor internalization) assays. For non-infectious diseases, for which protein targets are known, a target-based screening approach is very relevant. In contrast, targeting the causative infectious agent as a whole is an interesting approach for parasitic or other infectious diseases. Indeed, if one considers the field of antibacterials, target-based screens have been fairly unsuccessful in recent years [27].

We describe here the development of a new screening assay to identify new compounds active against *Leishmania*. This assay selects for a specific phenotype (absence or reduction of parasites in macrophage host cells), and this parameter is used to measure compound activity; all potential targets will thus be exposed to the tested compounds, thereby increasing the probability of finding active compounds with different modes of action in an optimized fashion.

Reporter genes have been successfully developed for anti-leishmanial HTS by various research groups [28], [29]. However, a major drawback of current reporter assay approaches was the need for continuous drug selection to maintain expression of the reporter gene over time. For *Leishmania*, this is particularly true for both episomal transient transfection and integrated transgenes, because the parasite has high genomic plasticity and a high recombination ratio [30]. Furthermore, the use of selection drugs can be problematic because they may directly or indirectly interact with the tested compound, interfering with the screening results. Recently, two independent research groups presented stable modified *Leishmania* parasites expressing GFP that are suitable for compound testing [31], [32]. Although engineered parasites are an invaluable tool in drug screenings, the use of wild-type organisms should be prioritized whenever possible because of potential for modifications in the general metabolism of the organism, due to expression of the transgene, resulting, for example, in a loss of virulence, as reported by de Toledo and collaborators [33] and as observed during this study with GFP-expressing *L. donovani* (data not shown). The use of wild-type parasites allows for the anti-leishmanial activity of compounds to be evaluated against different species of parasites, including clinical isolates, without the need for any genetic modification of these parasites. This approach required the development of a new tool for the image analysis of the parasite-infected cells: because DNA staining was the chosen method, the image analysis algorithm used had to identify each host cell as an isolated object, while also detecting intracellular parasites, all from the same image channel. Cell segmentation was the first challenge. By choosing the Voronoi diagram method, we were able to identify the inner boundaries of individual cells. The foreground mask applied to the Voronoi diagram image was then used to remove the background area, excluding all extracellular parasites from the analysis. The foreground mask was defined by pixels with intensity greater than 50. We observed that, given the stability of the image statistics across the screen and the good contrast between foreground and background, a fixed value was a robust way of separating foreground and background across the entire set of images. We tested several precise segmentation methods, such as region growing or a watershed algorithm, to measure the influence of the methods on the analysis results, and concluded that there was no significant difference in the infection ratio when precise segmentation methods or Voronoi diagrams were used. However, computational cost was much lower when we used the Voronoi diagram. In addition, *Leishmania* parasites could be detected inside the boundaries of each cell in the same image.

Image-based processing is a promising approach for chemical screening [34]. In our study, using a single channel for fluorescence labeling of both the host cells and parasites considerably simplified the technical complexity of the assay, but created a computational challenge for selectively classifying cells and parasites from the same fluorescence signal channel. An algorithm was developed and implemented to automatically process the readout images, allowing the analysis of the huge amount of data generated from the screens (Figs. 1 and 2).

The development of an accurate tool to quantify the infection ratio enabled the measurement and comparison of compound activity against the intracellular stage of the parasite. This result, in addition to validating the biological model by confirming that replicative intracellular *L. donovani* can infect a human macrophage cell line, establishes this system as the most relevant and promising for the discovery of new compounds for leishmaniasis treatment.

In addition to the infection ratio and parasite number, another parameter widely used to assess compound cytotoxicity is the number of host cells. Potentially toxic compounds that affect replication or cell multiplication will not be detected as toxic based on host cell counting because THP-1 cells usually do not replicate after differentiation, resulting in a constant cell number. However, compounds that induce necrosis or apoptosis or interfere with the adherence of the macrophages to the surface will be interpreted as toxic because of the low host cell number.

Ultimately, this newly developed standard protocol for the *Leishmania* infection model allowed the screening of 200,000 compounds at 20 µM for anti-parasitic activity in a high-throughput mode using optimal conditions, to be reported elsewhere. A subset of this library (26,500 compounds) has also been screened at 10 µM by our group against the promastigote form of *Leishmania* parasites in a fluorimetric assay [12]. Approximately 50% of the hits found in the intracellular amastigote assay were not found in the hit list obtained against the promastigote form. Conversely, only 4% of the hits from the promastigote screen were active against the intracellular amastigote. Even though the screenings used different species of parasites (*L. major* promastigotes and *L. donovani* intracellular amastigotes), we consider this difference relevant because we recently obtained similar data when screening a focused library of 4,000 compounds using only the *L. donovani* strain. These data are in accordance with recently published work from Muylder et al., which shows that using the promastigote form in a primary screen leads to a great number of hits that are likely to be inactive on the amastigote, and misses active compounds that are only found when using the intracellular amastigote [35]. Taken together, these results illustrate that a substantial number of compounds may be specifically active against intracellular amastigotes and would only be selected as true positive hits if tested in this screening system, supporting the proposal that the intracellular amastigote model is the most appropriate for drug discovery in leishmaniasis.

In conclusion, the development and validation of this HTS protocol for *Leishmania* infection of human macrophages without the need for a reporter gene is a major breakthrough in the field of leishmaniasis drug discovery. It fills a major gap and should allow the screening of diverse and focused compound library sets, opening up a new avenue for the identification of new compound series, which are critically needed to develop new drugs for the treatment of Leishmaniasis. The combination of a infection model with image-based analysis has proven to be a relevant method for screening compounds for their activity against the intracellular amastigote *Leishmania*.

Moreover, this protocol is well established for HTS and may be used on a smaller scale, such as in 96-well plates or even in lower throughput, in any research laboratory to test single compounds and/or natural product extracts.

## Supporting Information

Figure S1 ***Leishmania*** **parasites growth in different containers.** The graph contains a comparison of parasites growth pattern in T.175 flask (blue curve) and in Erlenmeyer bottle (red curve).
(TIFF)

Figure S2 **Effect of PMA concentration on the infection ratio.** The graphs represent the results of cell number (top left), parasite number (top right) and infection ratio (bottom right) after THP-1 host cells had been differentiated with different PMA concentrations. Large batch of cells and parasites (LB) simulating a screening run and small batch (SB), as used in pilot assay development, were used for comparison.
(TIFF)

Figure S3 **Methods for differentiated THP-1 cells release from culture flask.** The graphs show the results of number of parasites, number of cells, infection ratio and total number of infected cells obtained after the infection of THP-1 cells harvested from different methods from the T.175 flasks, and incubated with two different concentrations of PMA (20 ng/ml and 50 ng/ml).
(TIFF)

Figure S4 **Infection assay scale up.** Results comparing the infection ratio obtained from small scale and large scale experiments.
(TIFF)

Figure S5 **Screening standard operating protocol.** Flowchart illustrating the linear process of the assay from cell culture to data analysis. The steps in yellow are the ones performed manually and the steps in orange are performed by robots in an automated fashion.
(TIF)

Figure S6 **Comparison of the results of screening assays with promastigotes or intracellular amastigotes as the parasite model.** Selecting hits from screening using the promastigote form (insect) generated 124 hits, 5 of which were also active against intracellular amastigotes. Another screen using intracellular amastigotes in the primary assay generated 123 hits, 62 of which were also active against promastigotes.
(TIF)

Movie S1 **Three-dimensional reconstruction of a THP-1 cell infected with** ***L. donovani*** **.** The movie highlights replicating intracellular amastigotes (green) after BrdU incorporation assay. The blue color represents DNA staining (DAPI) and red represents host cell cytoplasm (as a background of Syto60 staining).
(AVI)
