# Supplementary figures and images for: An Image-Based High-Content Screening Assay for Compounds Targeting Intracellular Leishmania donovani Amastigotes in Human Macrophages
Source: PLoS Negl Trop Dis. 2012 Jun 12;6(6):e1671. doi: 10.1371/journal.pntd.0001671 (PMC3373640; doi:10.1371/journal.pntd.0001671)

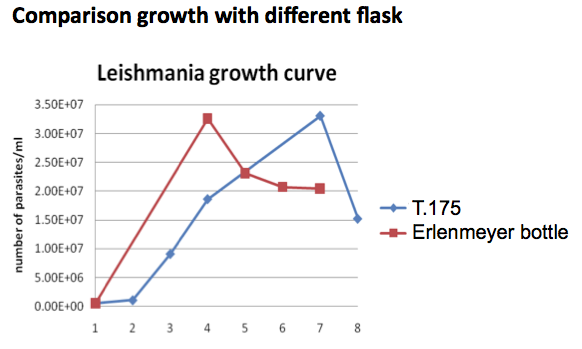

Supplement: Figure S1 — Leishmania parasites growth in different containers. The graph contains a comparison of parasites growth pattern in T.175 flask (blue curve) and in Erlenmeyer bottle (red curve). (TIFF) [file pntd.0001671.s001.tif]

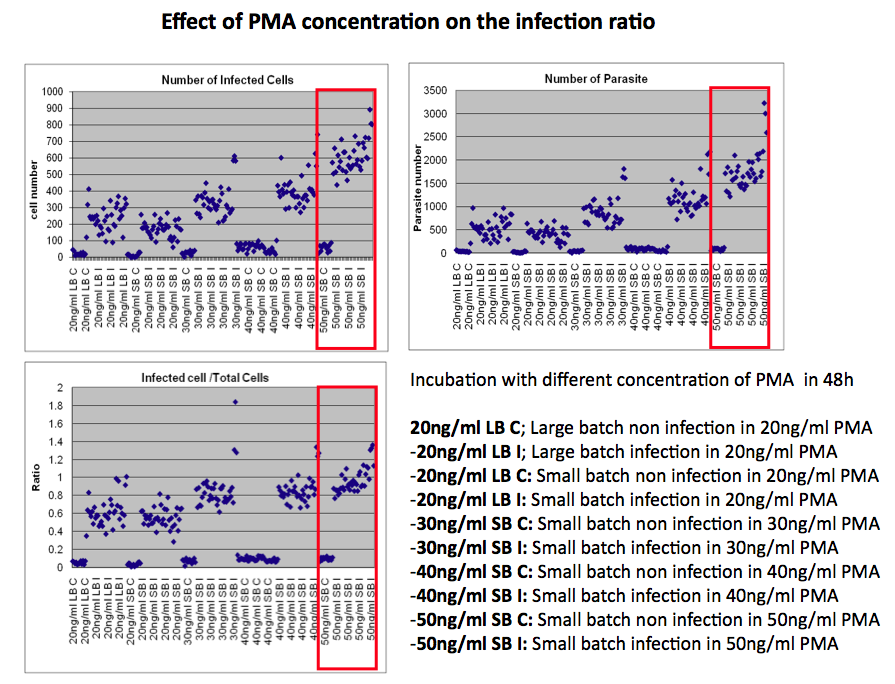

Supplement: Figure S2 — Effect of PMA concentration on the infection ratio. The graphs represent the results of cell number (top left), parasite number (top right) and infection ratio (bottom right) after THP-1 host cells had been differentiated with different PMA concentrations. Large batch of cells and parasites (LB) simulating a screening run and small batch (SB), as used in pilot assay development, were used for comparison. (TIFF) [file pntd.0001671.s002.tif]

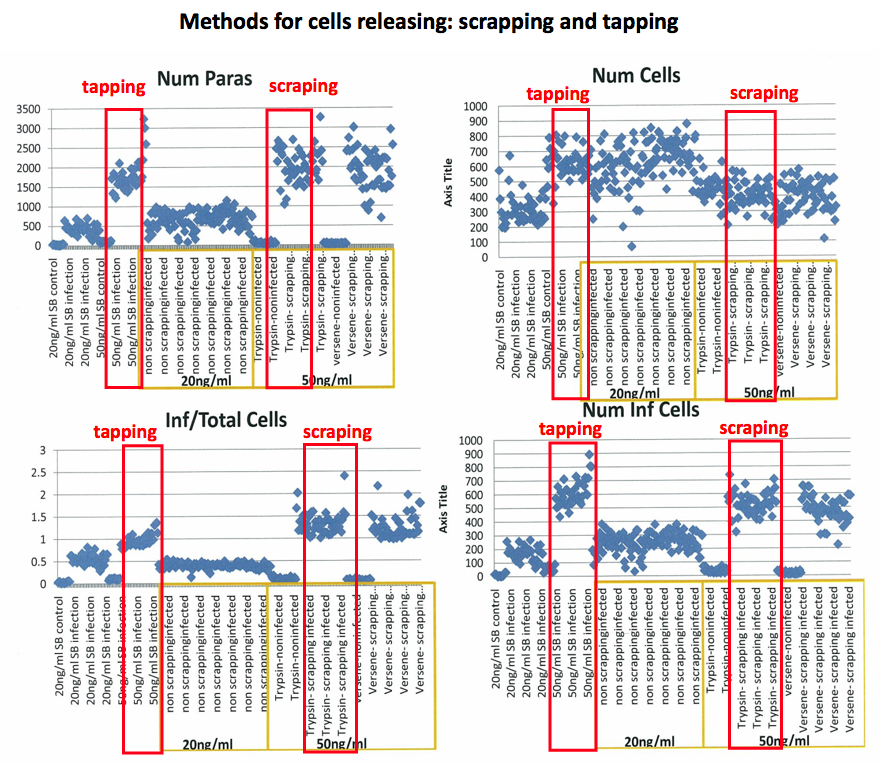

Supplement: Figure S3 — Methods for differentiated THP-1 cells release from culture flask. The graphs show the results of number of parasites, number of cells, infection ratio and total number of infected cells obtained after the infection of THP-1 cells harvested from different methods from the T.175 flasks, and incubated with two different concentrations of PMA (20 ng/ml and 50 ng/ml). (TIFF) [file pntd.0001671.s003.tif]

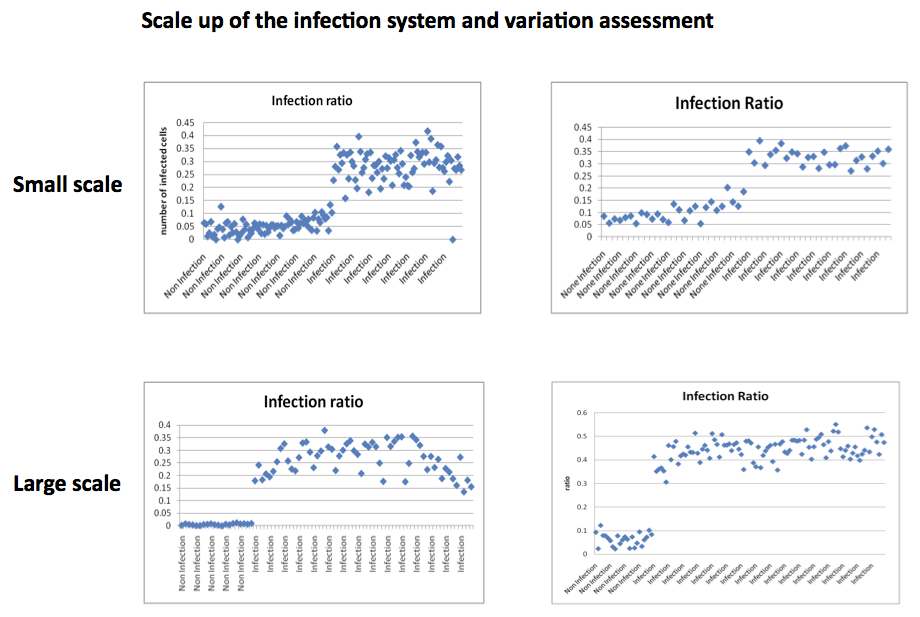

Supplement: Figure S4 — Infection assay scale up. Results comparing the infection ratio obtained from small scale and large scale experiments. (TIFF) [file pntd.0001671.s004.tif]

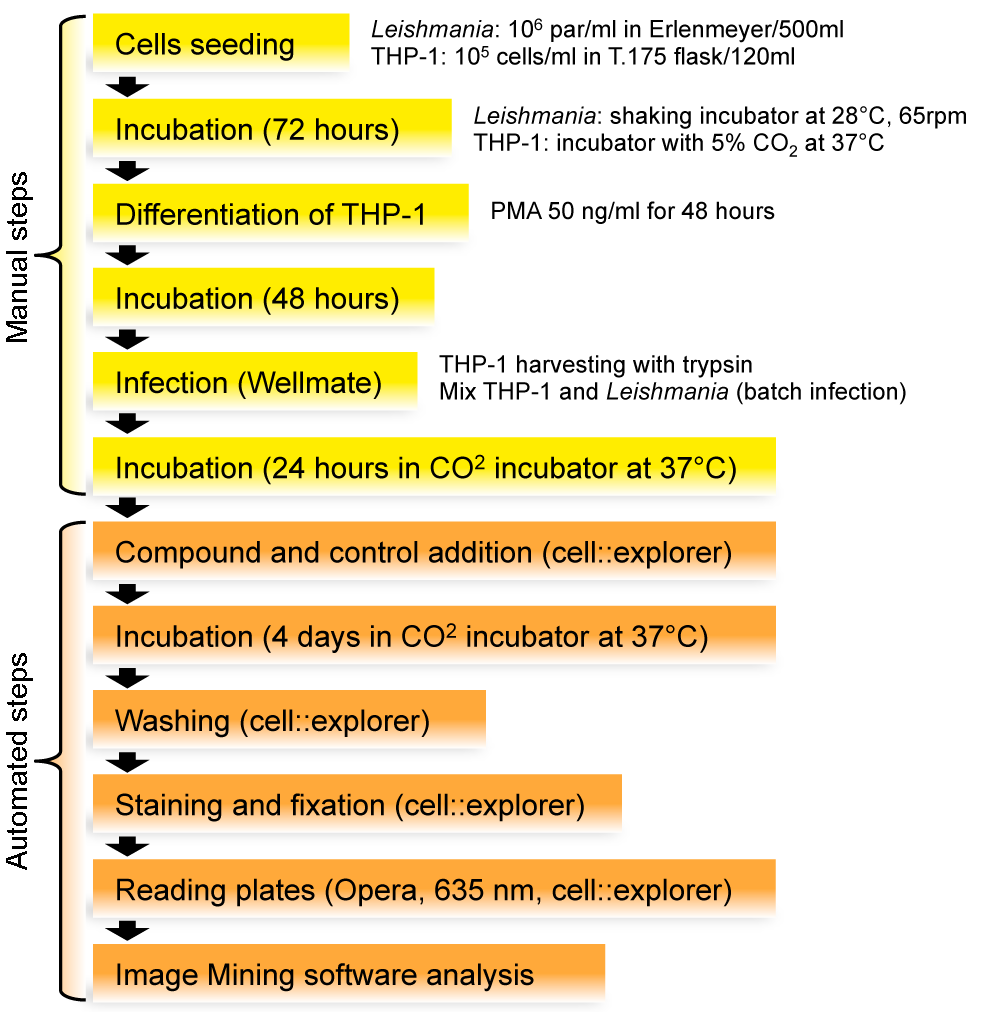

Supplement: Figure S5 — Screening standard operating protocol. Flowchart illustrating the linear process of the assay from cell culture to data analysis. The steps in yellow are the ones performed manually and the steps in orange are performed by robots in an automated fashion. (TIF) [file pntd.0001671.s005.tif]

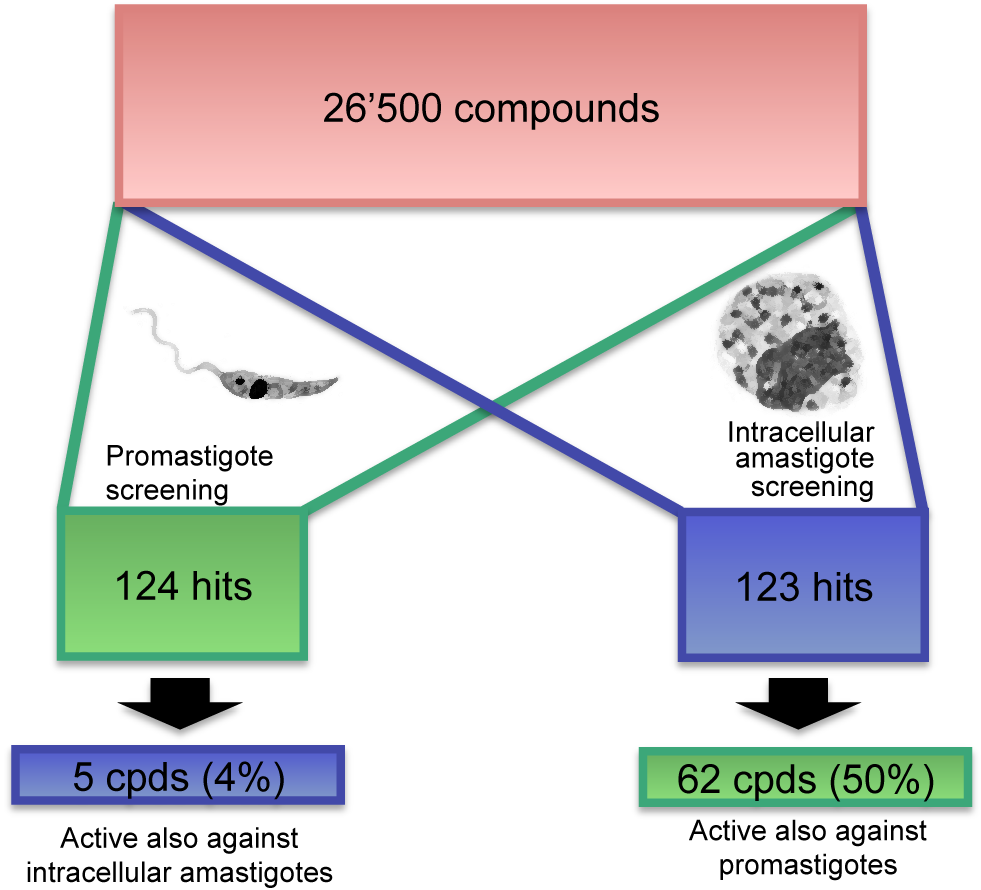

Supplement: Figure S6 — Comparison of the results of screening assays with promastigotes or intracellular amastigotes as the parasite model. Selecting hits from screening using the promastigote form (insect) generated 124 hits, 5 of which were also active against intracellular amastigotes. Another screen using intracellular amastigotes in the primary assay generated 123 hits, 62 of which were also active against promastigotes. (TIF) [file pntd.0001671.s006.tif]
